# Cannulation-related wound complications after extra corporeal life support: A retrospective cohort study in a Dutch intensive care unit

**DOI:** 10.1371/journal.pone.0337952

**Published:** 2025-12-22

**Authors:** Ineke van de Pol, Margreet van Rees, Gwen van Gool, Dewi Stalpers, Lisette Schoonhoven, Peter Noordzij, Erik Scholten

**Affiliations:** 1 Department of Intensive Care, St. Antonius Hospital, Nieuwegein, The Netherlands; 2 Department of General Practice & Nursing Science, Julius Center for Health Sciences and Primary Care, University Medical Center, Utrecht University, Utrecht, The Netherlands; 3 School of Health Sciences, Faculty of Environmental and Life Sciences, University of Southampton, The United Kingdom; 4 Department of Anaesthesiology and Intensive Care Medicine, University Medical Center Utrecht, Utrecht University, Utrecht, The Netherlands; CUNY School of Medicine: The City College of New York CUNY School of Medicine, UNITED STATES OF AMERICA

## Abstract

Extracorporeal life support (ECLS) is a high-risk therapy for acute cardiac or respiratory failure. After weaning, wound healing at the cannulation site is often disrupted, leading to discomfort, delayed recovery, increased nursing workload, and higher costs. Available data on this issue is limited. This study describes the epidemiology of cannulation-related wound complications (CRWCs) in ECLS patients, defined as impaired wound healing at the cannulation site >72 hours after decannulation. A retrospective, single-center cohort study was conducted in the Intensive Care Unit of St. Antonius Hospital in the Netherlands. Between 2018 and 2023, successfully weaned ECLS patients treated with venovenous (VV) or venoarterial (VA) ECLS for more than 24 hours were included. Descriptive statistics were used to describe the incidence and characteristics of CRWCs. Risk factors for CRWCs were assessed using univariate logistic regression at the patient and insertion-site levels. A total of 73 patients were included in this study, of whom 33 (45%) had 37 CRWCs. CRWCs were characterized by fluid leaks (90%), wound dehiscence (70%), tissue necrosis (76%), and/or wound infection (49%). CRWCs were primarily located at the groin and the first signs appeared after seven days (IQR 5–9 days). Identified risk factors included increased age, lower BMI, lower nadir serum albumin, VA cannulation, surgical insertion, and dual cannulation. Wound healing was still incomplete in 17 (51%) patients at the time of hospital discharge. CRWCs occur in nearly half of all successfully weaned VV and VA ECLS patients, mostly at the groin. Due to the severity of the patient’s illness and the acute clinical setting, the identified risk factors cannot always be avoided. This study raises awareness, aiding in the early and better identification of at-risk patients and recognizing a CRWCs as a serious complication as early as possible.

## Introduction

Extra Corporeal Life Support (ECLS) is a high-risk therapy for acute cardiac or respiratory failure when conventional treatments are insufficient. The number of patients who receive ECLS therapy is increasing, and 50–60% of these critically ill patients are successfully weaned from the ECLS device [1]. ECLS patients are at high risk of serious complications [2–4], including a cannula-related wound complication (CRWC). Due to the impaired healing of the wound, patients experience pain and discomfort and delayed recovery, resulting in longer hospital stays [5,6]. Nurses play an essential role in observing, preventing, and managing these complications [7,8].

CRWCs have an incidence ranging from 4.1% to 50% depending on the definition, patient population, cannula insertion site, and ECLS modality [5,6,9,10]. CRWCs are characterized by various clinical presentations, such as hematoma, wound dehiscence, vascular complications, wound infections, or fluid leakage or collection (i.e., seroma, lymphoceles, lymphatic leakage) [5,6,9,10]. Overall, wound infections are most frequently reported. CRWCs require complex wound care and often surgical interventions [5,11,12]. Due to the complexity and intensity of the necessary care, the nursing workload increases, as does the use of wound management materials. This increased need for care, higher material usage, and extended hospital stays contribute to higher healthcare costs.

In previous studies, several risk factors were identified, such as comorbidities, cannulation insertion site, ECLS configuration, and the duration of the ECLS run [5,6,10,11]. However, the available evidence is still limited. Most studies to date have mainly focused on venoarterial (VA) ECLS, often in highly selected patient groups such as cardiac transplant patients [5,6,10]. Findings from these populations may not be representative of the broader VA ECLS population, and data on venovenous (VV) ECLS patients are scarce. Therefore, we conducted an exploratory study to investigate the epidemiology of CRWCs occurrence in a more heterogeneous ECLS population. This study describes the incidence and characteristics of CRWCs in both VV and VA ECLS patients and examines whether these complications are confined to VA ECLS or also occur in VV ECLS. In addition, we analyzed potential risk factors for impaired wound healing across the entire study population. By providing a broader perspective, this study contributes to raising awareness on this topic, which helps in better identifying at-risk patients and recognizing a CRWC as early as possible.

## Materials and methods

### Study design, setting, and population

This single-center, retrospective cohort study involved adult ECLS patients at St. Antonius Hospital in the Netherlands. The hospital does not have a transplant facility. All patients who received VV or VA ECLS therapy for more than 24 hours between January 1, 2018, and December 31, 2023, were included. Exclusion criteria included cannulation at a hospital other than St. Antonius, death during ECLS therapy, or loss to follow-up (i.e., early post-decannulation mortality or transfer to another hospital). Patients who died during ECLS were excluded, because they were never decannulated and therefore could not develop a CRWC, which by definition in our study could only occur after decannulation.

The study was approved by the review board of the local ethics committee, and the requirement for informed consent was waived (Medical Research Ethics Committee United, reference number W22.204). Patients who had recorded an objection to the use of their healthcare data for scientific research in their medical records were excluded from the study.

### ECLS-care

ECLS cannulas were inserted at different locations in the hospital depending on ECLS indication or urgency. During each insertion, cannulation was performed under sterile conditions and guided by ultrasound. After insertion, the cannulas were secured using a purse-string or cross-stitch suture, as well as a Hollister StatLock fixation device. Subsequently, the insertion site was marked to facilitate position checks, and a transparent dressing was applied. In VV ECLS patients, the cannulas were inserted percutaneously. In VA ECLS patients, the cannulas were inserted either percutaneously or surgically. Standard distal limb perfusion was prophylactically created via an antegrade femoral arterial catheter (AFC). The venous cannulas ranged from 19 to 25 French, while the arterial cannulas were primarily 21 or 22 French, both being High-Flow System (HLS) cannulas (Getinge).

Patients receiving ECLS therapy (Cardiohelp system, Getinge) were cared for by a team of ECLS-trained nurses, perfusionists, and ICU doctors in the ICU. Every shift, a checklist was completed to assess the system, circulation in the legs, and the cannulation site. The assessment of the cannulation site included checking for correct insertion depth, leaks of exudate or blood, and ensuring that dressings were adequate. To prevent blood clots in the ECLS circuit, all patients received continuous intravenous heparin infusion. In addition, all patients were treated with selective digestive decontamination (SDD), including intravenous cephalosporins. Antibiotic prophylaxis was also given during ECLS support. Some awake patients mobilized despite ECLS therapy. However, this applied to only a small number of individuals.

Patients were decannulated after completing the VV or VA ECLS weaning protocol. In general, percutaneously inserted cannulas were removed in the ICU by an ICU doctor. A purse-string suture was tightened, and after manual compression, a compression device (FemoStop or Safeguard) was applied if necessary. The insertion site at the jugular vein could only be manually compressed. Surgically inserted cannulas were removed under direct visualization, using a prosthesis or patch if needed, by a thoracic or vascular surgeon. After decannulation, the care involved monitoring for and treating any potential bleeding. The sutured wound was covered with a regular dressing, and stitches were removed after 5–7 days.

### Outcomes parameters

Cannulation-related wound complications after ECLS were defined as the primary outcome and were assessed by a specialized wound care nurse. All patients were evaluated using the Wound Care Scale (WCS) classification model based on the TIME framework (Tissue, Inflammation/Infection, Moisture, Edge). Blinding of the assessor was not feasible, as access to the patient’s medical records was required to accurately assess the presence or absence of CRWCs. A CRWC was defined as impaired wound healing at the cannulation site more than 72 hours after decannulation. A threshold of 72 hours after decannulation was applied to define a CRWC, as normal physiological wound healing processes typically initiate within this period, while signs of abnormal wound healing, such as infection, usually become clinically apparent only after three to seven days. Impaired wound healing was characterized by excessive fluid leakage (blood/exudate), wound dehiscence, fibrinous slough, tissue necrosis, and/or confirmed wound infection. A wound infection was diagnosed when common pathogens/species responsible for wound infection were found in a wound culture: *Staphylococcus aureus*, coagulase-negative *staphylococci*, *Escherichia coli*, *Enterococcus faecalis*, *Pseudomonas* spp., *Enterobacter* spp., and *Klebsiella* spp., and *Proteus spp.*.

Secondary outcomes included interventions for CRWCs, such as treatment with antibiotics, negative pressure wound therapy (VAC), surgery, consultation of a specialized wound nurse, or intensive wound care (e.g., alginate-based dressing, bacterial-binding dressing, multifunctional dressing, or hydrofiber dressing). The duration of complete wound healing was also recorded.

### Risk factors

Candidate risk factors were selected after a literature search and consultation with ECLS experts. The factors were divided into three categories:

- Patient-related: gender, age, Body mass index (BMI), comorbidities (i.e., diabetes, chronic kidney disease, peripheral vascular disease) [5,6,10]
- Disease-related: APACHE IV, SOFA score, vasoactive agents, renal replacement therapy (RRT), nadir serum albumin concentration [5,10]
- ECLS-related: ECLS indication, ECLS configuration, insertion location, cannula insertion site, cannula vessel, cannulation duration, decannulation procedure, complications during the run (e.g., dislocation or blood leaks) [5,6,10,11]

### Data collection

Patient data (e.g., gender, age, BMI, comorbidities, APACHE IV score, ICU treatment, and serum albumin) and details about the ECLS run (e.g., indication, configuration, cannula specifications, and complications), as well as CRWC specifications, were collected from the electronic patient records (EPIC Systems Corporation).

Data extraction was retrospective and was performed by a medical student and the second author (patient characteristics and the ECLS run) and by a specialized wound nurse (CRWC specifications).

Where possible, data were extracted from structured electronic medical record (EMR) fields using predefined data queries. This applied to all quantitative variables such as patient demographics and treatment characteristics. In contrast, data related to the assessment of CRWCs were obtained through manual chart review across multiple documentation fields, as these data were not available in a standardized or structured format. This data collection took place between March 1, 2023, and March 1, 2024. Data management was conducted by the first and second authors, while data analysis was performed by the first author. Study data were managed using REDCap (Research Electronic Data Capture), hosted at the St. Antonius Hospital [13,14]. REDCap is a secure, web-based software platform designed to support data capture for research studies. The 1st, 2nd, and 3rd authors had access to information that could identify individual participants during or after data collection, as they had access to a key file. All data were anonymized before analysis and stored securely to protect patient confidentiality.

### Statistical analysis

Descriptive statistics were reported, using means with standard deviation (sd) and medians with interquartile range (IQR), as appropriate for the distribution of the variables.

All patients were divided into two groups: those with a CRWC and those without. Statistical analyses used the chi-square test or Fisher’s exact test for categorical variables, and the independent t-test or Wilcoxon’s rank-sum test for continuous variables to compare the two cohorts. Analyses were performed in the overall cohort, without stratification by ECLS configuration, due to the limited sample size. However, cannulation configuration (VA vs VV) was included as a covariate in the risk factor analyses. In addition to analyses at the patient-level, a range of variables – including cannulation insertion site and duraction – were assesed at the insertion-site level. Candidate risk factors with p-values <0.05 were included in a univariate regression analysis to identify their potential associations (RR).

Missing data were anticipated for variables such as APACHE IV score, SOFA score, cannula size, decannulation procedure, and wound treatment. Variables with less than 20% missing data were considered sufficiently complete and included in the analysis. Variables with more than 20% missing data were excluded to maintain data integrity and avoid biased estimations.

Statistical analysis was performed using R software, version 4.4.0.

## Results

### Study population

**Fig 1:**
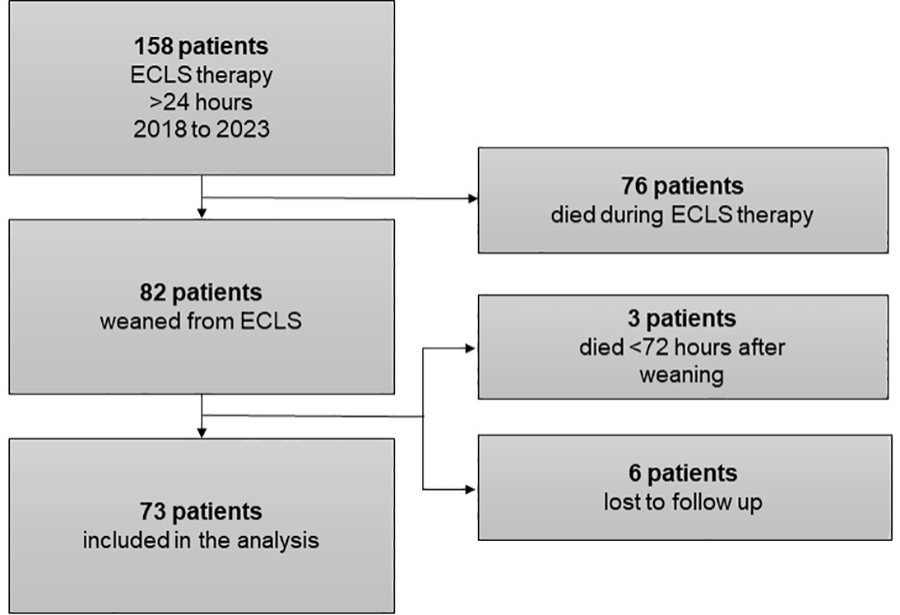
Patient selection.

Between 2018 and 2023, 158 ECLS patients were included, of whom 82 (52%) patients were successfully weaned. Nine patients (11%) were excluded due to death (n = 3), or lost to follow-up (n = 6). Data from 73 ECLS patients were analyzed. None of the patients had multiple ECLS runs (Fig 1).

Most patients were male, the median age was 58 years (IQR 48–70), and they had a median APACHE IV score of 81 (IQR 63–102) (Table 1). The ECLS indication was often after cardiac or lung surgery, with VA cannulation being the most common configuration, and a median ECLS duration of six days (IQR 4.5–10.0).

**Table 1:** Patient and ECLS characteristics.

	No CRWC n = 40	CRWC n = 33	p-value
Male (n, %)	25 (63.0)	19 (57.6)	0.669
Age, years (median, IQR)	54.0 (46.5–64,5)	65.0 (50.0–72.0)	0.024
Body Mass Index (median, IQR)^a^	28.6 (26.3–32.7)	26.0 (24.5–29.2)	0.012
Comorbidities (n,%) - Diabetes - Chronic kidney disease - Peripheral vascular disease	3 (7,5) 4 (10.0) 0 (0.0)	7 (21.2) 5 (15.2) 3 (9.1)	0.169 0.723 0.088
APACHE IV (median, IQR)^b^	77.0 (62.8–103.0)	82.5 (71.5–99.8)	0.477
Mechanical ventilation, days (median, IQR)^c^	13.0 (9.0–17.0)	20.0 (10.0–31.0)	0.101
Renal replacement therapy (n, %)	14 (35.0)	20 (60.6)	0.031
Vasoactive agents (n,%) - Pre ECLS run - During ECLS run	34 (85.0) 35 (87.5)	31 (93.9) 33 (100.0)	0.280 0.060
Nadir serum albumin concentration, g/L (mean, sd)	20.1 (5.3)	16.8 (5.3)	0.009
ECLS indication (n, %) - Post-thoracic surgery - Cardiac - Respiratory	14 (35.0) 8 (20.0) 18 (45.0)	20 (60.6) 10 (30.3) 3 (9.1)	0.003
ECLS configuration (n, %) - venovenous (VV) - venoarterial (VA)	20 (50.0) 20 (50.0)	6 (18.2) 27 (81.8)	0.013
ECLS duration, days (median, IQR)	5.5 (4.3–9.7)	6.7 (4.6–11.0)	0.495

^*a*^*2NA,* ^*b*^*15 NA,* ^*c*^*3NA.*

In these 73 patients, a total of 164 cannulas (venous and arterial) were inserted at 135 different anatomical sites, mostly in the groin. Among all cannulas, 38% were inserted percutaneously, whereas the remainder were placed surgically. Dual cannulation was observed in 29 cases, indicating that two cannulas were inserted through the same anatomical site (Table 2). During the ECLS runs, partial cannula displacement occurred in three patients.

**Table 2:** Insertion site characteristics (n = 135).

	No CRWC n = 98	CRWC n = 37	p-value
Cannula insertion site (n, %) - Jugular vein - Groin: Femoral vein or artery - Subclavian artery - Central	29 (29.6) 42 (42.9) 4 (4.1) 23 (23.5)	0 (0.0) 36 (90.0) 1 (10.0) 0 (0.0)	<0.001
Percutaneous insertion (n, %)^a^	45 (33.6)	6 (4.5)	0.003
Cannula vessel (n, %) - Artery - Vein - Dual; artery and vein in same site	19 (19.4) 65 (66.3) 14 (14.3)	2 (5.4) 20 (54.1) 15 (40.5)	0.002
Cannula duration, days (median,IQR) - Artery - Vein	5.1 (3.6–7.2) 6.3 (4.6–10.2)	4.1 (2.8–6.7) 6.7 (4.1–13.0)	0.367 0.768

^a^ *1 NA.*

### Cannulation-related wound complications

**Fig 2:**
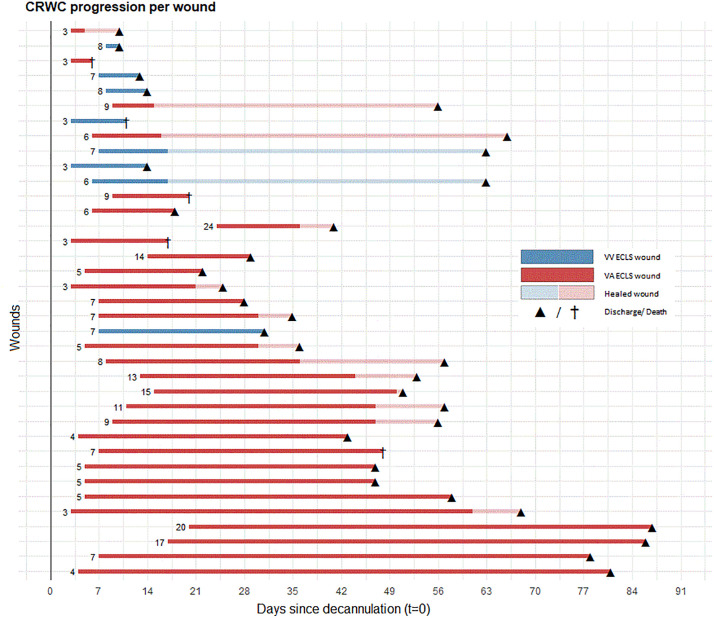
Timing and progression CRWCs.

Out of 73 ECLS patients, 33 (45%) had a CRWC**.** Four patients had multiple CRWCs, resulting in a total of 37 CRWCs. In VV ECLS patients, the incidence was 23% (6/26), while in VA ECLS patients, the incidence was higher at 57% (27/47). CRWCs were primarily located at the groin and the first signs appeared after seven days (IQR 5–9 days). See Fig 2 for the timing and progression of the wound complication, stratified by VV and VA ECLS patients. The most common clinical symptoms were leaks of exudate, and fibrinous slough or necrosis (Table 3). A cannula site wound infection was found in 49% of the wounds.

**Table 3:** Outcomes CRWCs.

Clinical symptoms and impact	n = 37
Days from decannulation to onset of wound complication (median, IQR)	7 (5.0–9.0)
Leaks after decannulation (n, %) - Blood - Exudate - Pus	8 (21.6) 33 (89.2) 15 (40.5)
Dehiscence (n, %)	26 (70.3)
Fibrinous slough or necrosis (n, %)	28 (75.7)
Cannula site wound infection (n, %)	18 (48.6)
Interventions at the wound level (n, %) - Wound materials (alginate based dressing, bacterial binding dressing (Sorbact®), multifunctional dressing (Polymem®), hydrofiber dressing (Aquacel®)) - Negative pressure wound therapy (VAC) - Surgical intervention Interventions at the patient level (n, %) - Antibiotics - Specialized nurse on consultation	25 (67.6) 2 (5.4) 10 (27.0) 13 (39.4)^a^ 21 (63.6)^a^
Completed healing process before discharge hospital (n, %)	16 (53.2)

^*a*^ *in 33 patients.*

These CRWCs required complex wound care, antibiotics and surgical interventions. For more than half of the wounds (77%), intensive wound therapy with multiple wound materials were used. Negative pressure wound therapy was initiated for two CRWCs (5%), and a surgical intervention was performed for ten wound complications (27%). In 13 patients (39%), antibiotics were started for an infected wound. In 21 patients (64%), a specialized wound nurse was consulted. At the time of hospital discharge, wound healing was complete in nearly half of the patients.

The length of stay in both the ICU (31 vs 20 days, p = 0.015) and the hospital (51 vs 33 days, p = < 0.006) was longer in the CRWC group. The in-hospital mortality was similar between the two groups (no CRWC: 23% vs CRWC: 18%, p = 0.65).

### Risk factors

Patients with CRWCs were older (RR 1.034, 95% CI 1.004–1.067, p = 0.030) and had a lower BMI (RR 0.969, 95% CI 0.937–0.995, p = 0.032) than those without CRWCs. During ECLS support renal replacement therapy (RRT) (RR 1.765, 95% CI 1.075–2.375595, p = 0.031) and a lower nadir serum albumin concentration (RR 0.971, 95% CI 0.946–0.993, p = 0.013) were associated with a higher incidence of CRWCs. VA cannulation increased the risk for a CRWC (RR 2.498, 95% CI 1.404–3.510, p = 0.006).

At the insertion-site level, most CRWCs occurred at the groin site, which was associated with an increased risk of CRWCs (RR = 26.308, 95% CI = 8.457–53.551, p < 0.001). Percutaneous insertion was protective compared with surgical insertion (RR = 0.325, 95% CI = 0.123–0.686, p = 0.003). In addition, the presence of two cannulas, dual cannulation, at a single insertion site was associated with an increased risk of wound complications (RR = 2.492, 95% CI = 1.499–3.474, p = 0.001).

## Discussion and conclusions

This study described the epidemiology of CRWCs in a single centre general ECLS population and found an incidence of 45%. CRWCs were primarily located at the groin site and characterized by fluid leaks, tissue necrosis, infection and wound dehiscence, and required complex wound care, antibiotics, and surgical interventions. In half of the CRWC patients, wound healing was still incomplete at the time of hospital discharge.

In literature, the incidence of CRWCs at the groin site ranges from 29% to 50%, depending on the patient population and ECLS modality, which corresponds with our results [5,6,9,10]. This variation mainly reflects cohort differences, with rates up to 50% in heart transplant patients [5] versus around 30% in broader VA-ECLS populations [6,9]. However, there is a lack of evidence regarding the VV ECLS group, as most studies focus on VA ECLS patients. In our cohort, the incidence of CRWCs especially in the VV ECLS group was significantly lower than in VA ECLS patients, yet still 30%. It remains unclear whether the cannula blood vessel site (i.e., venous, arterial) influences CRWC development. Safaya et al. (2024), demonstrated that dual cannulation at the same groin may increase the risk of CRWCs [15,16], which is also recognized in our data. Reported incidences also differ because studies use varying time windows for complication assessment, ranging from the acute ECLS phase to late post-decannulation [6,9–11]. Notably, in our study the first signs of a CRWC appeared at one week after decannulation, whereas in other studies symptoms typically arise between 22–25 days post-transplant or ECLS decannulation [5,10]. However, very late complications, occurring 50–150 days after discharge, have also been reported [11]. A CRWC has various clinical manifestations, often presenting in combination. It is not always clear whether a symptom is the cause or the result, such as in the case of a wound infection. Most studies report a comparable spectrum of complications, such as wound infections, fluid leaks or collection, hematomas, lymphoceles, or wound dehicence [5,6,9,10]. Isath et al. [5] particularly emphasized infected groin wounds following VA-ECLS decannulation, while Roussel et al [9], and Banks et al [11] also decribe vascular complications after ECLS. Similar to the studies by Isath et al. (2023) and Smood et al. (2023), our cohort’s CRWC patients had a longer average hospital stay [5,6].

ECLS therapy is known to be high-risk due to potentially severe complications during the run [3,4,16,17]. However, a serious complication like CRWC can also occur after ECLS therapy. Since this late complication develops only afterward, it may no longer be directly linked to the therapy, which could contribute to a lack of awareness. The longer hospital stay for patients with a CRWC suggests that recovery for these patients takes longer than patients without a CRWC. The development or non-development of a CRWC has never been reported in previous ECLS studies where length of hospital stay was the outcome. It is possible, that the occurrence of a CRWC could have been an unmeasured confounder in these studies and possibly influenced the outcome.

Univariable analyses suggested associations of older age, lower BMI, VA ECLS, RRT, and hypoalbuminemia with CRWCs. The univariate association of lower BMI with CRWCs contrasts with Ohira et al., who reported higher infection rates in patients weighting >100 kg [10]. Regarding insertion-site variables, groin cannulation was associated with an increased risk of CRWCs in our study, consistent with earlier reports [5,6,9,10], whereas cannula duration showed no such association [11]. In contrast, percutaneous cannulation appeared to be protective compared with surgical insertion, which is also consistent with findings reported in the literature [18].

The choice of cannulation technique and anatomical insertion site depends on the indication, the severity of the patient’s illness, and the acute circumstances at the time of cannulation, and is therefore not always modifiable. Therefore, we can better focus on improving the recognition of patients at-risk for a CRWC and the adequate treatment of these wound complications. The ICU nurse plays an important role in this process, as they are holistically trained to provide comprehensive patient care, including wound management, while also understanding concepts related to ECLS therapy [7]. At our center, cannulation and wound care practices are already highly standardized, which may explain why modifiable risk factors did not emerge as significant in our analysis. Nonetheless, it remains important to critically evaluate the current cannula care, from insertion to removal. Although the Extracorporeal Life Support Organization (ELSO) has numerous guidelines, including ECLS cannulation procedures, cannula care guidelines are lacking. Standardizing cannula and cannula site care during and after the ECLS support could potentially help reduce the occurrence of CRWCs.

The strength of this study was that it examined CRWCs in the entire ECLS population, rather than just a specific group. Additionally, our study contributes to raising awareness of this topic, which helps to better identify at-risk patients and recognize a CRWC as a serious complication as early as possible. This awareness should initially focus on the intensive care unit but must also be effectively extended to the general wards, as CRWCs can sometimes develop at a later stage after decannulation.

The limitations of this study include its retrospective design and small sample size, potentially leading to several methodological issues. First, the percentage of missing data for individual risk factors ranged from 4% to 25%. SOFA scores and data related to cannula size and removal were excluded from the analysis because the proportion of missing data exceeded 20%, reducing their reliability and interpretability. Second, due to the small sample size, multivariable regression could not be performed, which may have resulted in biased risk estimates and a lack of correction for confounding factors. The associations identified through univariate analyses at the patient and insertion-site levels should be interpreted with caution and further investigated in future studies using multivariable models at the patient level and multilevel analyses to account for multiple insertion sites per patient. Lastly, due to limited standardization in ECLS care accross centers, this single-center study has reduced generalizability because of the high level of variability in practice. In future, it is important to validate the results of this study in a larger and multicenter cohort, and add a focus on the different methods of cannula care. Additionally, it would be valuable to assess the impact of a CRWC on the patient, nursing workload, and costs in a future study.

CRWCs occur in nearly half of all successfully weaned VV and VA ECLS patients. But despite this high incidence, it is a relatively unknown complication. It is important to improve the identification of at-risk patients and to detect and manage CRWCs early and adequately. Gaining evidence or insights into critical care management in ECLS patients might optimize outcomes in these patients.

## References

[1] Wheeler CR, Bullock KJ. Extracorporeal membrane oxygenation. Respir Care. 2023;68(8):1158–70. doi: 10.4187/respcare.10929 37402582 PMC10353178

[2] Lorusso R, Belliato M, Mazzeffi M, Di Mauro M, Taccone FS, Parise O. Neurological complications during veno-venous extracorporeal membrane oxygenation: does the configuration matter? A retrospective analysis of the ELSO database. Crit Care. 2021;25(1):15. 33731186 10.1186/s13054-021-03533-5 PMC7968168

[3] Lorusso R, De Piero ME, Mariani S, Di Mauro M, Folliguet T, Taccone FS, et al. In-hospital and 6-month outcomes in patients with COVID-19 supported with extracorporeal membrane oxygenation (EuroECMO-COVID): a multicentre, prospective observational study. Lancet Respir Med. 2023;11(2):151–62. doi: 10.1016/S2213-2600(22)00403-9 36402148 PMC9671669

[4] Aubron C, DePuydt J, Belon F, Bailey M, Schmidt M, Sheldrake J, et al. Predictive factors of bleeding events in adults undergoing extracorporeal membrane oxygenation. Ann Intensive Care. 2016;6(1):97. doi: 10.1186/s13613-016-0196-7 27714705 PMC5053950

[5] Isath A, Gregory V, Ohira S, Levine A, Dhand A, Laskowski I, et al. Groin wound management after decannulation of veno-arterial extracorporeal membrane oxygenation in heart transplantation: role of sartorius muscle flap. Clin Transplant. 2023;37(12):e15101. 10.1111/ctr.15147 37755149

[6] Smood B, Fowler C, Rao SD, Genuardi MV, Sperry AE, Goel N, et al. Subacute groin complications related to ECMO cannulation are associated with longer hospitalizations. J Artif Organs. 2023;26(2):119–26. 35751721 10.1007/s10047-022-01342-3

[7] Parrett M, Yi C, Weaver B, Jones M, Almachar MB, Davidson J. Nursing roles in extracorporeal membrane oxygenation: a successful ECMO program needs a strong care team. Am J Nurs. 2024;124(11):24–32. 10.1097/01.NAJ.0001081100.87718.df 39446511

[8] Van Kiersbilck C, Gordon E, Morris D. Ten things that nurses should know about ECMO. Intensive Care Med. 2016;42(5):753–5. doi: 10.1007/s00134-016-4293-8 26957080

[9] Roussel A, Al-Attar N, Alkhoder S, Radu C, Raffoul R, Alshammari M, et al. Outcomes of percutaneous femoral cannulation for venoarterial extracorporeal membrane oxygenation support. Eur Heart J Acute Cardiovasc Care. 2012;1(2):111–4. doi: 10.1177/2048872612449417 24062897 PMC3760531

[10] Ohira S, Dhand A, Hirani R, Martinez S, Lanier GM, Levine A. Cannulation-related adverse events of peripheral veno-arterial extracorporeal membrane oxygenation support in heart transplantation: Axillary versus femoral artery cannulation. Clin Transplant. 2023;37(3):e15003. doi: 10.1111/ctr.15003 36468757

[11] Banks CA, Blakeslee-Carter J, Nkie V, Spangler EL, Still SA, Eudailey KW, et al. Occurrence, predictors, and management of late vascular complications following extracorporeal membrane oxygenation. J Vasc Surg. 2024;79(3):864-72.e1. 10.1016/j.jvs.2024.04.041 38657701

[12] Winiszewski H, Boyadjian C, Besch G, Soumagne T, Jeanney M, Pili-Floury S. Extracorporeal membrane oxygenation cannula-related infections: epidemiology and risk factors. ASAIO J. 2022;68(4):571–6. 34074852 10.1097/MAT.0000000000001505

[13] Harris PA, Taylor R, Minor BL, Elliott V, Fernandez M, O’Neal L, et al. The REDCap consortium: Building an international community of software platform partners. J Biomed Inform. 2019;95:103208. doi: 10.1016/j.jbi.2019.103208 31078660 PMC7254481

[14] Harris PA, Taylor R, Thielke R, Payne J, Gonzalez N, Conde JG. Research electronic data capture (REDCap)--a metadata-driven methodology and workflow process for providing translational research informatics support. J Biomed Inform. 2009;42(2):377–81. doi: 10.1016/j.jbi.2008.08.010 18929686 PMC2700030

[15] Safaya A, Yang S, Giglia JS, Moura Leite JO. Ipsilateral dual cannulation is associated with wound complications following veno-arterial ECMO decannulation. J Cardiovasc Surg (Torino). 2024;65(3):296–301. doi: 10.23736/S0021-9509.24.12874-1 39007557

[16] Simons J, Di Mauro M, Mariani S, Ravaux J, van der Horst ICC, Driessen RGH, et al. Bilateral femoral cannulation is associated with reduced severe limb ischemia-related complications compared with unilateral femoral cannulation in adult peripheral venoarterial extracorporeal membrane oxygenation: results from the extracorporeal life support registry. Crit Care Med. 2024;52(1):80–91. doi: 10.1097/CCM.0000000000006040 37678211

[17] Lorusso R, Barili F, Mauro MD, Gelsomino S, Parise O, Rycus PT, et al. In-hospital neurologic complications in adult patients undergoing venoarterial extracorporeal membrane oxygenation: results from the extracorporeal life support organization registry. Crit Care Med. 2016;44(10):e964-72. doi: 10.1097/CCM.0000000000001865 27340754

[18] Danial P, Hajage D, Nguyen LS, Mastroianni C, Demondion P, Schmidt M, et al. Percutaneous versus surgical femoro-femoral veno-arterial ECMO: a propensity score matched study. Intensive Care Med. 2018;44(12):2153–61. doi: 10.1007/s00134-018-5442-z 30430207

